# Association of chorioamnionitis with failed conversion of epidural labor analgesia to cesarean delivery anesthesia: A retrospective cohort study

**DOI:** 10.1371/journal.pone.0250596

**Published:** 2021-05-05

**Authors:** Yumi Katakura, Yusuke Nagamine, Takahisa Goto, Hiroyuki Sumikura

**Affiliations:** 1 Department of Anesthesiology and Critical Care Medicine, Yokohama City University Hospital, Fukuura, Kanazawa-ku, Yokohama, Kanagawa, Japan; 2 Department of Anesthesiology, Juntendo University Hospital, Hongo, Bunkyo-ku, Tokyo, Japan; University of Insubria, ITALY

## Abstract

**Aim:**

This study aimed to examine the association between clinically diagnosed chorioamnionitis and failed conversion of epidural labor analgesia to cesarean delivery anesthesia.

**Methods:**

This retrospective, single-center cohort study, conducted in a university hospital, enrolled term parturients undergoing emergency cesarean section after induction of epidural labor analgesia between September 2015 and May 2019. For the purpose of this study, all cases were re-examined to ensure that they fulfilled the criteria of chorioamnionitis, regardless of the actual indication for cesarean section proposed by obstetricians at the time of application. The primary outcome was failure of conversion of epidural labor analgesia to cesarean delivery anesthesia. Multivariable logistic regression analysis was performed to investigate the association between chorioamnionitis and failure of anesthesia for cesarean section.

**Results:**

Among the 180 parturients reviewed, 58 (43.9%) fulfilled the criteria for chorioamnionitis. Failure of epidural conversion in the chorioamnionitis (+) group was significantly higher than in the chorioamnionitis (-) group (46.6% [27/58] vs. 18.9% [14/74], crude odds ratio = 3.7, 95% confidence interval: 1.7–8.3). After adjustment for potential confounders (age, body mass index, multiparity, and duration for epidural labor analgesia), chorioamnionitis was found to be associated with failure of anesthesia for cesarean sections (adjusted odds ratio = 3.6, 95% confidence interval: 1.6–8.4).

**Conclusions:**

Chorioamnionitis is associated with the failed conversion of epidural labor analgesia to cesarean delivery anesthesia.

## Introduction

Epidural labor analgesia is widely practiced worldwide, and in the USA, more than 70% of pregnant women opt for it [1]. However, it has been reported that 4–14% of pregnant women who had received labor analgesia needed cesarean sections after induction of epidural labor analgesia [2]. When an emergency cesarean section is proposed for a parturient who is receiving epidural labor analgesia, epidural analgesia can be converted to operative anesthesia by injecting local anesthetics through the existing epidural catheter. This process is often referred to as epidural conversion [3] and it is thought to be an advantage of having epidural labor analgesia. In the case of failed epidural conversion, however, general or spinal anesthesia may be required. Therefore, the risk factors of failed conversion of epidural labor analgesia to cesarean delivery anesthesia have been widely studied [3–10].

Chorioamnionitis (CAM), a common indication for non-elective cesarean section during labor, is an infection of the chorion and/or amnion, and it activates the maternal and fetal inflammatory response systems. We hypothesized that activation of the inflammatory response may lead to increased activation of pain during labor and may cause difficulty in achieving adequate labor analgesia and operative anesthesia in patients with CAM [11]. However, the association between CAM and failed conversion of epidural labor analgesia to cesarean delivery anesthesia has not been studied.

The objective of this retrospective cohort study was to examine the association between CAM and failed conversion of epidural labor analgesia to cesarean delivery anesthesia.

## Materials and methods

### Design and setting

This single-center, retrospective cohort study was conducted in Juntendo University Hospital, a teaching hospital where 1,200 deliveries, including 850 deliveries receiving epidural labor analgesia, are performed annually. We collected data from medical records, anesthetic records, labor analgesia records, and partographs.

### Participants

From September 2015 to May 2019, term parturients who were aged > 20 years and who underwent cesarean sections while receiving labor analgesia were enrolled in this study. We excluded those parturients who required stat cesarean sections (category 1, National Institute for Health and Care Excellence [NICE] classification [12]), those who underwent surgery within 2 h of induction of epidural labor analgesia, and those who were not given any local anesthetics via the epidural catheter because of the subjective definition of the anesthesiologist, choosing an alternate anesthetic route for the provision of operative anesthesia.

### Standard protocol for labor epidural analgesia

Although this was a retrospective study, our standard protocol for labor analgesia and the anesthetic management of cesarean sections for parturients after the induction of labor analgesia were as follows. During the study period, all cases were managed under the supervision of our senior staff. Obstetric anesthesiologists performed all the anesthetic procedures in this study. The standard methods used for the induction of labor analgesia are combined spinal and epidural anesthesia (CSEA), dural puncture epidural (DPE), or epidural anesthesia (EPI). Anesthesiologists decided on the labor analgesia induction technique to be used. Labor analgesia was initiated in all women upon request, regardless of the degree of cervical dilation. For the induction of CSEA, 10 μg of fentanyl and 2.5 mg of bupivacaine were administered intrathecally via the L3–‍L4 interspace. Subsequently, an epidural catheter was placed using the through-the-needle technique. The DPE technique is a modification of the CSEA technique, where a dural perforation is created with a spinal needle without intrathecal medication [13]. For the induction of EPI and DPE, levobupivacaine (0.125%) with fentanyl (5 μg/mL) was administered incrementally (10–20 mL). For maintenance of labor analgesia, 0.1% ropivacaine or 0.08% levobupivacaine, with fentanyl (2 μg/mL), was administered using a patient-controlled epidural analgesia (PCEA) device regardless of the induction technique. Settings for PCEA were a bolus dose of 5 mL, a lockout time of 15 min, and no background infusion. Pain intensity was measured using numerical rating scales ranging from 0 to 10 points (0 = no pain, 10 = worst pain). Scores of ≤ 4 were considered to indicate adequate pain relief. Anesthesiologists assessed the block adequacy every 1–2 hours during labor analgesia, even with PCEA. When the decision was made to perform a cesarean section, 10 mL of 2% lidocaine and 1 mL of 7% bicarbonate were administered, and another 5 mL of 2% lidocaine with 100 μg of fentanyl was added when needed.

### Exposure

All cases during the study period were examined if they fulfilled the criteria of CAM, regardless of the proposed indication for cesarean section for the purpose of this study. We defined CAM as suspected Triple I [14] (intrauterine infection or inflammation, or both; S1 Table). The criteria are as follows.

Fever without a clear source plus any of the following:

1. baseline fetal tachycardia (> 160 bpm) for 10 min or longer, excluding accelerations, decelerations, and periods of marked variability;
2. maternal WBC > 15,000 per mm^3^ in the absence of corticosteroids; or
3. definite purulent fluid from the cervical os.

We collected laboratory data, symptoms, and vital signs from delivery records and electronic medical records, and the parturients whose data were complete for the criteria were classified into the CAM (+) group. All the parturients were retrospectively classified into the CAM (+) or CAM (-) group.

### Outcome

The primary outcome was the failure to convert epidural labor analgesia to cesarean delivery anesthesia. We defined this failure as the inability of anesthesiologists to provide adequate anesthesia until the end of surgery using only local anesthetics via the epidural catheter inserted for epidural labor analgesia and/or the need to administer a sedative agent or intravenous analgesics. We also investigated the ratio of conversion to general anesthesia in cesarean section.

The secondary outcomes included the following variables regarding labor analgesia; the number of PCEA requests that the parturient made by pushing the PCEA button, number of PCEA bolus administrations, hourly consumption of the PCEA solution, and the number of rescue boluses administrations by anesthesiologists against breakthrough pain. In addition, we checked the intervertebral range of ‘loss of cold’ level from L3/4.

### Covariates

We listed age, body mass index (BMI), whether the patient was parous or not, and the duration between the induction of labor analgesia and entry into the operating room as potential confounders.

The degree of urgency and the type of anesthesia were considered potential effect modifiers. We excluded the cases of stat cesarean section (category 1 of the NICE classification); therefore, the degree of urgency was unified. The types of anesthesia were divided into subgroups (CSEA or non-CSEA). The non-CSEA subgroup was too small to analyze; therefore, the results of the CSEA subgroup analysis are shown in supplementary tables. We categorized some quantitative variables including age, BMI, and the duration of anesthesia into groups during the analysis.

### Statistical analysis

Statistical analysis was performed using the JMP software package (JMP 12 pro; SAS Inc., Cary, NC, USA). The results were expressed as median (interquartile range [IQR]) or count (%). Univariate analysis was performed using the χ^2^ or Fisher’s exact test for categorical variables and Wilcoxon rank-sum test for continuous variables. Multivariable logistic regression was used to estimate multivariable-adjusted odds ratios (ORs) and 95% confidence intervals (CIs). The multivariable analysis was adjusted for age, BMI, multiparity, and duration of epidural labor analgesia. We considered the type of anesthesia as an effect modifier and analyzed the association between factors and outcomes as a subgroup analysis. Statistical significance was set at two-tailed p-values of less than 0.05.

### Ethical considerations

This study was approved by the ethics committee of Juntendo Hospital (18–223). This retrospective study employed opt-out consent. Information about research objectives, types of data to be collected, protection of personal information, and conflict of interest are made available on the web of Juntendo University Hospital. Opportunity to withdraw consent is provided on the web. The ethics committee approved the opt-out consent procedure. All data were fully anonymized, and data range was September 2015 to May 2019.

## Results

**Fig 1:**
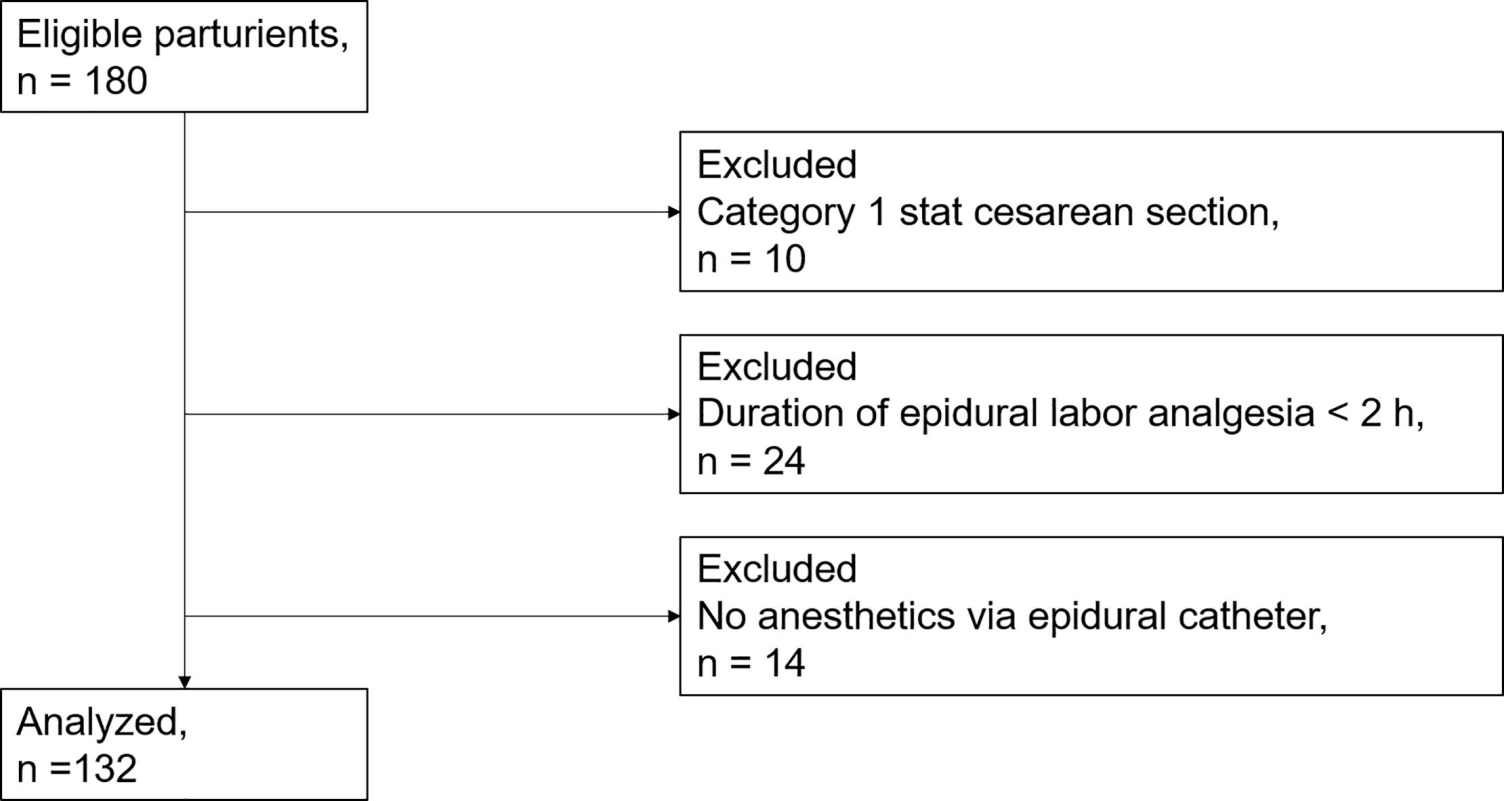
Study flow chart.

The study flow diagram is shown in Fig 1. During the study period, 180 parturients were enrolled. We excluded 10 parturients who underwent stat cesarean section (category 1), 24 who underwent cesarean section within 2 h after induction of labor analgesia, and 14 who were anesthetized for the operation without adding any local anesthetics via the epidural catheter because of an unreliable epidural catheter. The remaining 132 women were analyzed for this study. They underwent cesarean sections because of a non-reassuring fetal status or arrest of labor.

Table 1 shows the baseline characteristics of the study population. Among them, the median age was 36 years, and the median BMI was 25.0 kg/m^2^. The median gestational age was 40 weeks. Five participants were parous, and 10 parturients were positive for maternal group B Streptococcus status. A total of 58 parturients (43.9%) fulfilled the criteria for CAM. The BMI were significantly higher in the CAM (+) than the CAM (-) group.

**Table 1:** Baseline characteristics of the study population.

Characteristic	CAM (-)	CAM (+)	p-value
	n = 74	n = 58	
Maternal age (years)^a^	37 (33–40)	35 (34–39)	p = 0.53
< 35^b^	24 [32.4]	17 [29.3]	
35 ≤ y < 45^b^	49 [66.2]	40 [69.0]	
≥ 45^b^	1 [1.4]	1 [1.7]	
Parous women^b^	3 [4.1]	2 [3.4]	p = 0.86
Gestational age (weeks)^a^	40 (39–41)	40 (39–41)	p = 0.14
Pre-pregnancy BMI (kg/m^2^)^a^	20.4 (19.1–22.6)	21.2 (19.5–24.6)	p = 0.08
BMI (kg/m^2^)^a^	25.0 (22.0–26.3)	25.0 (23.8–30.0)	p = 0.04
BMI^b^ ≥ 25	42 [56.8]	41 [70.7]	p = 0.11
Maternal GBS status positive^b^	7 [9.5]	3 [5.2]	p = 0.36
GBS^b^ (missing)	4 [5.4]	5 [8.6]	p = 0.47

^a^Continuous variables are presented as median with (interquartile range).

^b^Categorical variables are presented as number [%].

Abbreviations: CAM: Chorioamnionitis; BMI: Body mass index; GBS: Group B Streptococcus.

Table 2 shows the anesthetic management of epidural labor analgesia of the study population. CSEA was the analgesic method used in 85.6% (113) of the parturients. Among them, the median duration of epidural labor analgesia was 8.0 h (IQR; 5.0–17.0). The duration of epidural labor analgesia was significantly longer in the CAM (+) than the CAM (-) group (11.5 h vs. 8.0 h). Decision-to-delivery time was defined as the duration from deciding on a cesarean delivery to the delivery time. Decision-to-delivery time was significantly longer in the CAM (+) than the CAM (-) group.

**Table 2:** Anesthetic management of epidural labor analgesia of the study population.

Parameter	CAM (-)	CAM (+)	p-value
	n = 74	n = 58	
Induction technique of labor analgesia			p = 1.00
CSEA^a^	64 [86.5]	49 [84.5]	
DPE^a^	7 [9.5]	6 [10.3]	
EPI^a^	3 [4.1]	3 [5.2]	
Duration of epidural labor analgesia (h)^b^	8.0 (4.5–14.0)	11.5 (6.0–19.0)	p = 0.03
< 10^a^	45 [60.8]	25 [43.1]	
10 ≤ h < 20^a^	17 [23.0]	20 [34.5]	
20 ≤ h < 30^a^	7 [9.5]	8 [13.8]	
30 ≤ h < 40^a^	3 [4.1]	5 [8.6]	
≥ 40^a^	2 [2.7]	0 [0.0]	
Cesarean delivery			
Decision-to-delivery time (min)^b^	56.5 (41.0–72.5)	73.0 (60.0–90.5)	p<0.001
Operation time (min)^b^	45.5 (39.0–56.0)	52.4 (42.8–70.3)	p = 0.03

^a^Categorical variables are presented as number [%].

^b^Continuous variables are presented as median with (interquartile range).

Abbreviations: CAM: Chorioamnionitis; CSEA: Combined spinal and epidural analgesia; DPE: Dural puncture epidural analgesia; EPI: Epidural labor analgesia.

### Primary outcome

The failure rate of epidural conversion is shown in Table 3. The failure rate in the CAM (+) group was 46.6%, which was significantly higher than the 18.9% in the CAM (-) group (crude OR 3.7, 95% CI 1.7–8.3, p<0.001). In the CSEA subgroup analysis, the failure rate in the CAM (+) group was also significantly higher than that in the CAM (-) group (crude OR = 3.4, 95% CI 1.5–7.8, p = 0.003, S2 Table).

**Table 3:** Primary outcome; failure rate of epidural conversion.

Group	Failure rate of epidural conversion	Crude odds ratio (95% CI)	p-value
CAM (-)	14/74 (18.9%)	ref.	-
CAM (+)	27/58 (46.6%)	3.7 (1.7–8.3)	p<0.001

Abbreviations: CAM: Chorioamnionitis; CI: Confidence interval.

After adjusting for potential confounders, CAM was associated with the failure of epidural conversion to cesarean delivery anesthesia (adjusted OR 3.6, 95% CI 1.6–‍8.4, p = 0.002, Table 4). A longer duration of epidural labor analgesia was associated with the failure of anesthesia for cesarean section (adjusted OR 1.8, 95% CI 1.2–2.7, p = 0.004). In the CSEA subgroup analysis, CAM was again associated with anesthetic failure (adjusted OR 3.4, 95% CI 1.5–8.2, p = 0.005, S2 Table).

**Table 4:** Primary outcome; multivariable logistic regression analysis.

Parameter	Adjusted odds ratio (95% CI)	p-value
CAM	3.6 (1.6–8.4)	p = 0.002
Age^a^	1.9 (0.8–4.9)	p = 0.13
BMI^b^	0.9 (0.4–2.3)	p = 0.88
Multiparous women	0.6 (0.0–4.9)	p = 0.69
Duration of epidural labor analgesia^c^	1.8 (1.2–2.7)	p = 0.004

^a^Age was adjusted for categorical variables (< 35; 35 ≤ y < 45; ≥ 45).

^b^BMI was adjusted for categorical variables (< 25; ≥ 25).

^c^Duration of epidural labor analgesia was adjusted for categorical variables (< 10; 10 ≤ h < 20; 20 ≤ h < 30; 30 ≤ h < 40; ≥ 40).

Abbreviations: CAM: Chorioamnionitis; BMI: Body mass index; CI: Confidence interval.

The rate of conversion to general anesthesia in the CAM (+) group was 1.7%, which was not significantly different from the 1.4% in the CAM (-) group (crude OR 1.3, 95% CI 0.1–20.9, p = 1.00, S3 Table).

### Secondary outcomes

The results of the secondary outcomes are shown in Table 5. Among the study population, the median number of PCEA requests was 17.0. The median number of PCEA boluses was 12.0, while the median number of local anesthetics used per hour was 7.4 mL/h. The median number of intervertebral ‘loss of cold’ level was 6. The parturients in the CAM (+) group tended to push the PCEA button more often and received significantly more PCEA boluses, more rescue boluses by anesthesiologists against breakthrough pain, and a higher hourly dose of local anesthetics. At the same time, there was no significant difference in the ‘loss of cold’ levels in either group (Table 5). CSEA subgroup analysis showed similar trends (S4 Table).

**Table 5:** Secondary outcomes; quality of epidural labor analgesia.

Parameter	CAM (-)	CAM (+)	p-value
PCEA requests^a^ (n)	13.0 (6.0–30.5)	23.5 (8.0–41.5)	p = 0.06
PCEA boluses^a^ (n)	10.5 (5.8–22.0)	18.5 (7.0–34.0)	p = 0.01
Consumption of PCEA solution^a^ (mL/h)	6.8 (5.0–8.8)	7.9 (6.2–10.8)	p = 0.02
Number of rescue boluses against breakthrough pain^b^ (n)	0 (0–1) [0, 4]	0 (0–2) [0, 10]	p = 0.03
Intervertebral range of ‘loss of cold’ level^a^	6 (5–8)	6 (5–8)	p = 0.67

^a^Continuous variables are presented as median with (interquartile range).

^b^Presented as median with (interquartile range) and [minimum and maximum values].

Abbreviations: CAM: Chorioamnionitis; PCEA: Patient-controlled epidural analgesia.

## Discussion

In this retrospective cohort study, we examined the association between CAM and failed conversion of epidural labor analgesia to cesarean delivery anesthesia and found that CAM is associated with higher failure rates after adjusting for potential confounders. Regarding the secondary outcomes, parturients with CAM needed significantly more local anesthetics for epidural labor analgesia. To our knowledge, this is the first study demonstrating such an association.

To date, the risk factors for failed conversion of epidural labor analgesia to cesarean delivery anesthesia have been widely studied. Various maternal factors have been implicated in the failure of epidural conversions. These include a young age [4], height (over 167 cm) [5], higher BMI and weight [4], greater gestational age [4], factors of analgesia such as the number of episodes of breakthrough pain [4,6,7] and required boluses of analgesics, the duration of labor analgesia [7], urgency of cesarean delivery [8], and anesthetic factors such as epidural analgesia alone [7] or non-obstetric anesthesiologists controlling labor analgesia [6,9]. Of these factors, the most critical are “initiation of neuraxial labor analgesia by non-obstetric anesthesiologists,” “additional epidural boluses,” and “urgency of cesarean delivery” [3,10]. However, maternal infection during labor has not been considered as a risk factor.

The rate of failed conversion of epidural labor analgesia to cesarean delivery anesthesia has also been widely studied. The Royal College of Anaesthetists in the United Kingdom recommends a threshold of <1% conversion of neuraxial anesthesia to general anesthesia in elective cesarean deliveries and a threshold of <5% for emergency deliveries [15]. In previous studies, the ratio of conversion of neuraxial anesthesia to general anesthesia has been reported to be about 2.1–4.4% [3]. The higher failure rates we demonstrated in this study (18.9% in the CAM (-) and 46.6% CAM (+) group) can be accounted for by our much stricter criteria for failure, which was not limited to conversion to general anesthesia but included any use of drugs other than epidural local anesthetics. It should be noted that the rate of conversion to general anesthesia in this study was 1.7% in CAM (+) and 1.4% in CAM (-) group (S3 Table), both of which were within the range of conversion rates reported by others [3,15].

The secondary outcome in this study showed that parturients with CAM were also more likely to experience severe pain during labor analgesia, which is consistent with our primary outcome. Moreover, this result is consistent with one of the reported risk factors for failed conversion of epidural labor analgesia to cesarean delivery anesthesia, namely “additional epidural boluses” [3].

The ratio of CAM among patients requiring cesarean sections in this study was also high. We defined CAM as suspected Triple I, not as confirmed Triple I. In order to make a definitive diagnosis of CAM (confirmed Triple I), Triple I should be accompanied by objective laboratory findings of infection in the amniotic fluid or histopathological evidence of infection or inflammation or both in the placenta, fetal membranes, or the umbilical cord vessels (S1 Table) [14]. Clinical decision making should be considered before confirmed Triple I criteria are met. Therefore, we decided to use the suspected Triple I criteria. However, one of the limitations of this study is that some cases could not be diagnosed using the confirmed Triple I criteria because histopathological or culture tests were not performed. Recent study suggested that clinical signs are actually not accurate in diagnosing CAM at the histopathologic level [16].

The association between CAM and failure of epidural conversion may be explained as follows. First, the increased labor pain in a parturient with CAM may be explained by previous reports that indicate that CAM causes uterine tenderness [17,18], and that inflammatory mediators, such as TNF-α, released by this inflammation contribute to the severe pain [11,19]. Second, it may be explained by the attenuated effects of local anesthetics under inflammation [20]. There is a possibility of localized acidosis in patients with inflammation due to intrauterine infection, which may inhibit local anesthetic uptake into the cells, thereby resulting in poor efficacy of local analgesia [21,22]. Third, it may be explained by increased metabolism of local anesthetics or washout of local anesthetics by increased blood flow in hyperthermic conditions [23]. Further research is needed to understand the mechanism behind the association.

There was a significant difference between the groups regarding the duration of epidural labor analgesia and the decision-to-delivery time. Our study design limits the inference of any firm conclusion on the mechanisms of these differences; however, we speculate that parturients with CAM might have requested the induction of epidural labor analgesia in the earlier stage because of uterine tenderness. This might explain the longer duration of epidural labor analgesia in the CAM (+) than the CAM (-) group. The longer decision-to-delivery time may indicate the lesser urgency for cesarean section in the CAM (+) group.

This study had several limitations. First, individual anesthesiologists used their discretion to provide anesthesia for cesarean sections after assessing the efficacy of the epidural catheter. Patients had to be excluded if local anesthetics were not administered via the catheter. Second, there are several potential sources of bias in this study. This study was conducted at a single center with a high proportion of elderly primigravidae and parturients with CAM, which could have resulted in selection bias. Third, some of the enrolled parturients did not undergo blood sampling while febrile and subsequently CAM may have been misdiagnosed. Fourth, we did not have a definitive pathological diagnosis of CAM. However, the diagnostic criteria that we used are used in clinical practice as an indication of CAM to make decisions regarding treatment [14]. It is a future task to conduct an analysis with pathologically confirmed CAM-positive and CAM-negative patients. Fifth, since the sample size was small, the confidence intervals of the odds ratios of the failure rates were fairly wide.

## Conclusions

This retrospective cohort study showed that CAM is associated with the failure of anesthesia for cesarean sections via the epidural catheter. Further research is needed to understand its effects and the mechanism by which CAM might diminish the efficacy of epidural anesthesia.

## Supporting information

S1 Table Features of isolated maternal fever and Triple I with classification. (XLSX)

S2 Table Primary outcome; CSEA subgroup analysis. (XLSX)

S3 Table Rate of conversion to general anesthesia. (XLSX)

S4 Table Secondary outcomes; CSEA subgroup analysis. (XLSX)
